# Exploring mechanisms of fatigue during repeated exercise and the dose dependent effects of carbohydrate and protein ingestion: study protocol for a randomised controlled trial

**DOI:** 10.1186/1745-6215-15-95

**Published:** 2014-03-26

**Authors:** Abdullah F Alghannam, Kostas Tsintzas, Dylan Thompson, James Bilzon, James A Betts

**Affiliations:** 1 Human Physiology Research Group, Department for Health, University of Bath, Bath BA2 7AY, UK; 2 School of Life Sciences, Queen’s Medical Centre, Nottingham NG7 2UH, UK

**Keywords:** Nutrition, Recovery, Carbohydrate, Protein, Performance

## Abstract

**Background:**

Muscle glycogen has been well established as the primary metabolic energy substrate during physical exercise of moderate- to high-intensity and has accordingly been implicated as a limiting factor when such activity is sustained for a prolonged duration. However, the role of this substrate during repeated exercise after limited recovery is less clear, with ongoing debate regarding how recovery processes can best be supported via nutritional intervention. The aim of this project is to examine the causes of fatigue during repeated exercise bouts via manipulation of glycogen availability through nutritional intervention, thus simultaneously informing aspects of the optimal feeding strategy for recovery from prolonged exercise.

**Methods/Design:**

The project involves two phases with each involving two treatment arms administered in a repeated measures design. For each treatment, participants will be required to exercise to the point of volitional exhaustion on a motorised treadmill at 70% of previously determined maximal oxygen uptake, before a four hour recovery period in which participants will be prescribed solutions providing 1.2 grams of sucrose per kilogram of body mass per hour of recovery (g.kg^-1^.h^-1^) relative to either a lower rate of sucrose ingestion (that is, 0.3 g.kg^-1^. h^-1^; Phase I) or a moderate dose (that is, 0.8 g.kg^-1^.h^-1^) rendered isocaloric via the addition of 0.4 g.kg^-1^.h^-1^ whey protein hydrolysate (Phase II); the latter administered in a double blind manner as part of a randomised and counterbalanced design. Muscle biopsies will be sampled at the beginning and end of recovery for determination of muscle glycogen resynthesis rates, with further biopsies taken following a second bout of exhaustive exercise to determine differences in substrate availability relative to the initial sample taken following the first exercise bout.

**Discussion:**

Phase I will inform whether a dose–response relationship exists between carbohydrate ingestion rate and muscle glycogen availability and/or the subsequent capacity for physical exercise. Phase II will determine whether such effects are dependent on glycogen availability *per se* or energy intake, potentially via protein mediated mechanisms.

**Trial registration:**

ISRCTN87937960.

## Background

It is well established that during prolonged moderate to high intensity exercise, carbohydrate is primarily utilised to support energy metabolism due to its efficiency and expeditious availability to support the energetic requirements of such activities [1-3]. In humans, the majority of endogenous carbohydrate is stored as glycogen in the muscle and liver [4] with the capacity to sustain muscle contractions at the aforementioned exercise intensities being highly dependent on the availability of this glycogen at these sites [5,6]. The physiological mechanisms responsible for these observations appear to involve several inter-related factors including: maintenance of euglycaemia and an attenuation of central nervous system fatigue; glycogen sparing; and reduced exercise-induced strain [7].

Recovery following exercise is mainly determined by the time required to replenish the depleted glycogen stores and ingesting sufficient carbohydrate (6 to 10 grams of carbohydrate per kilogram of body mass per day) is necessary to restore both glycogen depots and the capacity for repeated physical exertion [8-10]. Conversely, when periods of recovery are limited (≤8 hours), such as various training and competition scenarios, neither of the aforementioned processes can be entirely restored [11]. This underpins the importance of identifying nutritional strategies to maximise recovery and restoration of exercise capacity in concurrence with manifestations of fatigue during a repeated exercise bout. To date, nutritional research concerning recovery from exercise has been based on the assumption that similar fatigue mechanisms will operate during repeated exercise within hours of an initial bout, so has understandably focused on strategies to accelerate muscle glycogen resynthesis. While much is now known about the amount, type and timing of nutrient intake that can facilitate muscle glycogen storage and/or the capacity for repeated physical exertion [11], little evidence exists to demonstrate a link between these variables.

When examining these variables independently, it appears that muscle glycogen resytnhesis is augmented during recovery as opposed to a placebo [12,13] and a dose–response relationship exists between carbohydrate intake and muscle glycogen resynthesis until reaching a threshold of 1.2 grams per kilogram of body mass per hour of recovery (g.kg^-1^.h^-1^) [11]. In addition to the amount of carbohydrate provided, muscle glycogen storage is primarily influenced by the energy content irrespective of the macronutrient composition [14]. Consequently, the glycogenic properties of carbohydrate may be related to the increased energy content or mediated metabolic responses (hyperinsulinaemia) rather than the presence of carbohydrate fraction *per se*. Therefore, several studies were implemented to explore the efficacy of mixed macronutrient ingestion during recovery and repeated exercise, particularly carbohydrate-protein supplementation [15]. The culmination of the results suggests that increasing the amount of carbohydrate is sufficient to maximise glycogen resynthesis rates and restore endurance capacity more completely [11], although some indicate a distinct advantage of protein co-ingestion [16-18]. Further investigation is required, particularly during running-based exercise, to elucidate the efficacy of protein co-ingestion on restoration of muscle glycogen during limited recovery and the potential ergogenic effects upon subsequent endurance capacity.

Based on the above information, increasing carbohydrate intake during short-term recovery can accelerate muscle glycogen resynthesis. Nevertheless, endurance capacity may not concurrently improve [19,20]. Only one investigation reported an enhanced capacity for repeated exercise following limited recovery in response to higher carbohydrate intake [21]. Few investigations have been conducted to examine the role of muscle glycogen during a subsequent exercise bout [17,22,23], and none examined muscle glycogen degradation during a repeated exhaustive exercise bout. In accordance, glycogen availability may not play a fundamental role in contrast to an initial exercise bout whereby fatigue is intimately associated with glycogen depletion [5]. Different fatigue mechanisms may take place, and muscle glycogen metabolism during a subsequent exhaustive exercise bout following short-term recovery remains to be examined.

### Trial objectives

This research trial will investigate the relationships between carbohydrate intake, muscle glycogen metabolism and exercise capacity during a repeated bout of physical exercise. It will explore whether the effect of carbohydrate ingestion is causal between muscle glycogen availability and/or endurance capacity, and if this relationship is linked to the carbohydrate fraction *per se* or simply to an energy intake surplus. Thus, a replacement of a fraction of the carbohydrate with a different macronutrient (whey protein hydrolysate) matched in energy content will be examined to assess if whey protein hydrolysate co-ingestion provides a distinct benefit over carbohydrates alone in relation to muscle glycogen storage and/or the capacity for subsequent exercise. Specifically, we aim to accomplish the following objectives: (i) to examine whether a dose–response relationship exists between rates of carbohydrate (sucrose) ingestion and muscle glycogen availability following short-term recovery and/or the capacity for subsequent exercise; and (ii) to explore the potential protein-mediated effects upon muscle glycogen resynthesis and/or the capacity for a subsequent exercise bout with the ingestion of whey protein hydrolysate.

The use of the disaccharide sucrose was chosen on the basis of its potential positive contribution to liver and/or muscle glycogen resynthesis by virtue of equimolar amounts of glucose and fructose. Following exhaustive exercise, sucrose and glucose ingestion seem to elicit similar muscle glycogen resynthesis rates [24]. However, resting intravenous [25,26] and oral ingestion [27] studies indicate that fructose preferentially stores liver glycogen relative to glucose, while glucose infusion favours muscle glycogen resynthesis. Given the importance of both liver and muscle glycogen replenishment during short-term recovery and subsequent endurance capacity [28], sucrose was deemed a preferable source of carbohydrate to undergo predominant hepatic metabolism (that is, fructose) to optimise liver glycogen resynthesis alongside a glucose source to maximise muscle glycogen storage [29].

An important factor determining the rate of muscle glycogen resynthesis is insulin-mediated glucose uptake by the muscle cells [8]. A proposed mechanism for the potential benefit of protein co-ingestion in enhancing the rate of glycogen storage is the synergistic effect of this substrate on insulin secretion [30,31]. It has been recently demonstrated that plasma insulin response increases to a greater extent in whey than in casein protein in its intact form [32]. The ingestion of a protein hydrolysate accelerates digestion and absorption compared with its intact protein, resulting in a more rapid increase in circulating insulin concentrations [33]. This was further confirmed by the finding of greater insulinotropic properties when whey protein hydrolysate was ingested as opposed to whey protein in humans [34].

Concerning glycogen storage, the ingestion of whey protein has been shown to stimulate this process more rapidly both in liver and skeletal muscle tissues than when casein was ingested [35]. Furthermore, it appears that ingesting whey protein hydrolysate with carbohydrate augments glycogen resynthesis to a greater extent than when carbohydrate is co-ingested with intact whey protein, casein or intact branched-chain amino acids [36]. Taken together, these results indicate that a hydrolysed whey protein fraction may have a profound role in stimulating insulin secretion and concomitant muscle glycogen storage, and thus forming the basis for the inclusion of this protein fraction in the current study.

### Trial design

The methods described here stem from a wider project titled ‘Macronutrient ingestion, Muscle glycogen and Post-Exercise Recovery’. Both trial phases described herein have been approved by the National Health Service (NHS) South West 3 Research Ethics Committee (REC) with an allocated reference number: 09/H0101/82. The project was subsequently registered as a clinical controlled trial (ISRCTN 87937960).

Phase I of testing will address objective (i) by way of a non-randomised repeated measures design. The chosen trial design for Phase I is based on the premise that a lower rate of sucrose ingestion will mediate sub-optimal recovery, thus resulting in impairment in exercise capacity relative to a higher rate of sucrose intake, as has been previously reported in a similar investigation [21]. It was, therefore, assumed that participants would run longer when the higher sucrose treatment was administered. Accordingly, a non-randomised design would allow not only an investigation of the metabolic environment at the point of fatigue during the low versus high sucrose treatments, but also at the time point in the high sucrose treatment coincident with the onset of fatigue during low sucrose treatment. Indeed, previous studies have adopted a similar approach to investigate mechanisms through which carbohydrate supplementation enhances the capacity for both cycling [37] and running [38] modes of exercise. In addition, participants will be fully familiarised with a trial (see experimental protocol) that will be identical to the main procedures and, therefore, diminishing any order effects. This will consequently improve the reliability of the performance measure that would enable the detection of small but worthwhile intervention effects [39].

The second phase (Phase II) of testing will address objective (ii) by adopting a randomised double-blind cross-over experimental design.

## Methods/Design

### Ethical considerations and safety

The individuals who agree to participate following the briefing will be provided with an informed consent form, indicating their full understanding of the study and their protected rights for confidentiality and withdrawal from the study without giving a reason. Thereafter, a compulsory medical health questionnaire will be completed by each participant to ensure the absence of any physical, haematologic, metabolic or any other health conditions deemed to pose a risk to the participant or bias towards the investigation. If any of the factors above were present, the volunteer would be deemed unfit to participate in the study and would consequently be excluded from taking part. Further health questionnaires and profile of mood state (POMS) questionnaire will be completed before each trial to ensure that participants are fit and able to take part in testing and to establish similar physical and mental engagements throughout the trials.

#### Experimental design

The global design of the trial includes comparisons of two nutritional interventions during each distinct phase of testing. A minimum of two weeks wash-out period will be allowed between each main trial to avoid any carry-over effects. The independent variable will be the precise nutritional intervention applied during the short-term recovery period. This involves the provision of 0.3 g.kg^-1^.h^-1^ and 1.2 g.kg^-1^.h^-1^ of carbohydrate in the form of sucrose solutions in Phase I of testing and a 1.2 g.kg^-1^.h^-1^ sucrose versus an 0.8 g.kg^-1^.h^-1^ sucrose + 0.4 g.kg^-1^.h^-1^ whey protein hydrolysate in Phase II of the project. All treatments will be provided in equal volumes (10 ml.kg^-1^.h^-1^) and will be matched in sodium and potassium composition. The test beverages will also be matched in flavour (vanilla extract). Sample analyses of both treatments underwent screening by an independent institution (HFL Sport Science, LGC Ltd., Teddington, Middlesex, UK) to confirm the absence of any contaminants such as banned anabolic steroids and stimulants including noradrenaline, Tetrahydrogestrinone (THG) and 3,4-methylenedioxymethamphetamine (MDMA). A full description of the treatment composition is provided in Table 1.

**Table 1:** Nutritional information of the supplements provided in phase I and phase II of the trial

	**Low sucrose**	**High sucrose**	**Moderate sucrose + whey protein hydrolysate**
Sucrose (g/L)	30	120	80
Lactose (g/L)	-	-	≤3.5^a^
Protein (g/L)	-	-	40
Fat (g/L)	-	-	≤2.2^a^
Sodium (g/L)	0.38	0.38	0.38
Potassium (g/L)	0.47	0.47	0.47
Calcium (g/L)	-	-	0.2
Magnesium (g/L)	-	-	0.01
Phosphorous (g/L)	-	-	0.12
Chloride (g/L)	-	-	0.15
Energy (kcal/L)	120	480	480

^a^Assay unable to detect values below this number. The caloric content for fat and lactose was therefore assumed negligible.

To assess human endurance capacity, the trial adopted an exercise time to exhaustion (TTE) as an outcome measure. Although it has been argued that time-trial (TT) is more relevant than TTE as performance measure, these theories are based on the assumption that the primary outcome variable is a reflection of what occurs during actual sporting performance. These assumptions are generally founded on two factors: (1) there are no real-world sporting events that are the equivalent of TTE, which require an individual to run until the point of exhaustion; and (2) TTE appears to be less reliable than TT in relation to a valid simulation that resembles actual performance in a given sport [40]. While applied sports science studies mimic ‘real-world’ sporting events and provide a useful tool in assessing performance in a sports context, this naturally constrains them to the rules and regulations of a given sport that may be changed or refined periodically.

If we wish to interpret the value of performance measures in ‘real-world’ events, TTE may arguably be more prevalent and have a more important bearing in the broader scale. In terms of prevalence, there are clearly more recreational exercisers than athletes, who may present a case that maintaining their running or cycling endurance capacities to be able to engage with other exercisers at the same intensity and for the duration of the activity (such as running outdoors with friends or maintaining the capacity to complete a 90 minute football match) is paramount. Furthermore, many athletes undertake exercise scenarios similar to TTE tests. For example, pace is often set by the fastest athlete with the majority of athletes attempting to sustain this pace for as long as possible before reducing their pace and consequently falling behind the group/athlete. With regard to importance, endurance capabilities defined as ‘the ability of a muscle group to sustain external forces for long periods of time’ are considered an integral component in improving the occupational tasks of military personnel [41]. This underscores the importance of endurance capacity over performance time in this population whereby completing a march as a unit is the goal and not performance at maximal individual capabilities of a set task, which could separate individuals and pose risks for those personnel. Certainly, scientific investigations that measure constants in nature (that is, establishing the mechanisms of fatigue) should not be overlooked.

It should also be recognised that TTE measures vary widely in the literature and the actual reliability will depend on the protocol, individual and laboratory environment and, critically, familiarisation should not be viewed as a ‘one size fits all’ view. Given the importance of familiarisation, it is acknowledged that TT carry greater reliability from the outset, particularly if using relevant athletes and repetitive familiarisation with TTE is to be avoided. However, we have demonstrated in our current project sufficient reliability (CV = 3.5%, based on TTE during the first run to exhaustion between trials) when participants are familiarised and attempts are made to help them gauge their level of fatigue (that is, use of our three-strikes run-walk-run approach to achieve a metabolic end point). Taken together, TTE measures in endurance-trained athletes who are familiarised with the protocol are adequate to elicit reliable and sensitive measures.

The view that TT is a better representation of human performance is of course a valid one, if the sole reason for a performance measure is to produce an outcome measure that can be directly extrapolated to a real-world event. Nonetheless, it should be appreciated that a large number of scientific investigations involve mechanistic variables of human performance and its limitations, be it physiological, metabolic or neuromuscular among others. Fatigue is a complex phenomenon with several contributing factors, such that fatigue may occur simultaneously in several loci in the human body with the relative underlying mechanisms likely to overlap and interact [42,43]. It is imperative, therefore, that to investigate this intricate behaviour, means of inducing volitional fatigue through TTE performance measures are important to be included in a controlled environment to establish mechanistic causes of performance limitations and the possible mediating influences of certain nutritional interventions in delaying the onset of fatigue in humans. Accordingly, given that the aims of the current project are mechanistic in nature provides a foundation for the adopted TTE outcome measure.

#### Participants

The chosen target population for the research trial will be healthy non-smoking recreationally active men and women who include endurance training in the form of running as a central component (≥2 hours/week) of their training regime. The chosen age range for participation will be 18 to 48 years old. The research trial aims to recruit sixteen participants (eight in each phase of testing) from the University of Bath campus and surrounding sporting clubs by way of public advertisement and personal communication. These individuals will all be trained (as evidenced both by their self-reported training volume and also measures of maximal oxygen uptake during preliminary testing). Upon volunteering, the participants will be initially briefed in writing followed by a verbal explanation of the protocol and the pre-requisites set for satisfactory inclusion on their first visit to the laboratories to ensure their full understanding before the investigation. The target population sample will be free from any condition that either poses undue personal risk or introduces bias into the experiment (as determined by each participant’s responses to baseline health screening). In relation to eumenorrheic female participants, all measurements will be conducted at least three and at most ten days after the onset of menses (that is, the follicular phase) to ensure low levels of circulating female hormones and, therefore, minimise any measurement errors associated with the menstrual cycle. Each participant will be required to attend the Physiology Research Laboratory at the University of Bath on four occasions. These include: (i) a preliminary visit; (ii) a familiarisation visit; and (iii) two main trials completed on separate days.

### Control measures

#### Standardisation of lifestyle

Over the 48 hours prior to the familiarisation trial, a weighed dietary record will be completed for the analysis of macronutrient composition and total daily energy intake. The same diet will then be replicated prior to any laboratory visit involving the main procedures. Participants will be required to abstain from alcohol consumption for 24 hours before any trial, while caffeine abstinence will be started at 17:00 on the day preceding any trial. The latter aims mainly to avoid any unnecessary side effects that may negatively influence performance as a result of adverse withdrawal from caffeine [44]. Approximately 12 hours before the familiarisation session and, subsequently, before the experimental trials, a standardised meal will be provided for each participant. This is aimed at minimising any within-subject variability in the nutritional status of each participant that is known to influence metabolism and exercise performance and, ultimately, may influence the outcomes of the study [44,45].

Participants will be required to complete an activity log in conjunction with their dietary control procedures discussed in the experimental protocol. The participants will be instructed to refrain from any strenuous physical training for 48 hours before any trial with any light-to moderate habitual training recorded for time, duration and mode of exercise before the familiarisation session. This procedure will be matched for ensuing trials. Standardisation of lifestyle will be retrospectively analysed for nutritional intake (via nutritional assessment software) and activity (minutes of exercise per day) to ensure sufficient control over the testing period.

#### Environmental measurements

Ambient temperature, humidity and barometric pressure will be monitored and recorded at 60 minute intervals throughout the trials using a portable weather station (WS 6730; Technoline, Berlin, Germany). The latter will be used to record atmospheric pressure to allow for corrections to standard volumes during expired gas analysis.

#### Experimental protocol

Upon arrival in the laboratory on the day of familiarisation and each main trial, participants will have fasted for a period of ≥10 hours. Participants will then provide further written confirmation of their consent to take part, before completing an assessment of their mood state. A baseline urine sample will be collected from the first void of the day (30 minutes prior to testing) to ensure adequate hydration followed by a baseline assessment of body mass. Thereafter, participants will be placed in a semi-supine position before a five minute resting sample of expired gases is collected using the Douglas Bag technique [46]. An indwelling cannula will then be fitted to an antecubital vein and a 10 ml baseline blood sample collected, with the cannula kept patent throughout trials by frequent flushing with isotonic saline. Participants will then begin running on the treadmill with a standardised five minute warm-up at 60% of maximal oxygen uptake ( $\bar{V}O_{2\max}$) before the intensity will be increased to 70% $\bar{V}O_{2\max}$, which will be sustained until volitional exhaustion. To gauge their relative levels of fatigue accurately, participants will be permitted to reduce the intensity (walking at 4.4 km.h^-1^) for two minute intervals on two occasions when they indicate they can no longer sustain the running speed. A 2 ml venous blood sample will be collected during each walking phase, followed by a return to the prescribed running speed at 70% $\bar{V}O_{2\max}$. Volitional exhaustion will be accepted on the third occasion when participants indicate they are unable to exercise at the prescribed running intensity, and TTE will be recorded (excluding the time permitted for walking). To ensure the attainment of a metabolic endpoint, at least one of the predetermined criteria must be observed: (1) a reduction in blood glucose concentration below 3.9 mmol.L^-1^ and/or (2) the inability to sustain exercise following walking on two occasions. This is aimed at ensuring similar liver glycogen depletion levels during treatments and, thus, reducing the variability in carbohydrate availability during recovery. Immediately after this first run, participants will rest in a semi-supine position while a 3 to 5 mm skin incision will be made to the anterior portion of the thigh under local anaesthetic and a muscle biopsy obtained from the *vastus lateralis* using the percutaneous needle biopsy technique. This biopsy will be obtained in all treatment arms of the research project with the exception of the high sucrose treatment in Phase I. The biopsy will be utilised to compare possible mechanisms of fatigue during the subsequent bout of exercise between low and high sucrose treatments (further details provided below).

At this point the four hour post-exercise recovery will commence with the first volume of prescribed solution (10 ml.kg^-1^.h^-1^) ingested as soon as the first muscle biopsy has been removed from the leg, with the remaining seven aliquots provided at 30 minute intervals during the ensuing recovery. Participants will be permitted 15 minutes to consume each volume such that feeding will be complete 15 minutes prior to the second muscle biopsy. Expired gas and venous blood samples will be taken during the recovery period every hour prior to feedings. In addition, each participant’s total urine output will be collected during the recovery period and stored in a vessel containing 5 ml 10% thymol isopropanol as a preservative. Approximately 20 minutes prior to the end of recovery, a further two to three separate skin incisions will be made to the same leg and the second muscle biopsy will be sampled at four hours following the first sample. The remaining incision site(s) will then be available for further biopsies during/after the second exercise bout.

After the standardised warm-up, participants will again run on the treadmill at 70% $\bar{V}O_{2\max}$ until the point of volitional exhaustion. The achievement of a metabolic endpoint will be determined in a similar manner to the initial exercise bout. As in the first run, water intake will be *ad libitum* during the familiarisation trial and matched for the ensuing main trials. Blood will be obtained every 10 minutes for the first 30 minutes and at the point of volitional fatigue. Expired gas samples will be collected at 15 minute intervals and at the point of fatigue (that is, a final minute expired gas sample). In relation to Phase I, it is highly likely that all participants will exercise for longer in their second trial (that is, with the 1.2 g.kg^-1^.h^-1^ sucrose solution), so the exercise test will be briefly interrupted for an additional muscle biopsy at the point where fatigue occurred in each participant’s first trial (that is, with the 0.3 g.kg^-1^.h^-1^ sucrose solution). A further blood and gas sample will also be obtained during this time point to investigate the metabolic environment across treatments. Final blood and gas samples will be taken within the final minute of exercise and the remaining biopsy site will be used to obtain a post-exercise muscle sample. The three biopsies taken in any given trial will be sampled from the same leg, each separated by approximately 3 cm, with the use of dominant/non-dominant legs being counterbalanced between trials.

### Measures

#### Anthropometry

The determination of post-void nude body mass for the participants will be recorded on their first visit to the laboratory to allow for an accurate nutrient provision during subsequent visits that are calculated relative to each participant’s body mass. Measurements of body mass will also be required pre- and post-exercise on all ensuing visits to assess the hydration status of each participant via the recordings of the difference in nude body mass (reported as percentage difference) at the beginning and end of an exercise session. Weighing of each participant’s body mass will be undertaken by using a balance scale, where participants will be asked to stand in the centre of the platform while standing aligned with their weight evenly distributed on both feet. Measurement of the stature of the participants will be recorded by using a stadiometer. The participants will be requested to remove any footwear and to hang their arms freely with the palms facing the thighs and heels. It will be ensured that the gluteal area and shoulders make contact with the stadiometer to maintain sufficient alignment. To achieve accurate measurements, participants will be verbally advised to stand up straight while facing directly with the head in the Frankfort plane (orbitale and tragion are horizontally aligned). The participant inspires for measurement, and the recorder brings down the headboard to compress the hair [47]. From the collective measurements obtained above, body mass index can be determined (kg.m^-2^) for each participant.

#### Preliminary measurements

The preliminary tests to be administered will require approximately 60 to 90 minutes and involve an assessment of running economy (that is, $\bar{V}O_{2}$.km^-1^) and $\bar{V}O_{2\max}$[48]. These tests initially require participants to run on a motorised treadmill (Ergo ELG70, Woodway, Weil am Rhein, Germany). The protocol involves a standardised five minute warm-up that consists of jogging at a speed of 7.5 km.h^-1^, followed by a running economy test. This requires participants to run at various sub-maximal speeds with increments of 1 km.h^-1^ to enable a minimum of four different running speeds. Each running speed will consist of three minute stages, during which expired gas, heart rate (HR) and ratings of perceived exertion (RPE) will be recorded at the final minute of each stage. Expired gas, HR and RPE will be obtained via the Douglas bag method, short range telemetry (FT2, Kempele, Finland) and Borg’s (6 to 20) scale [49], respectively.

Approximately 20 minutes following the sub-maximal test, a run time to volitional exhaustion will be initiated to determine relative values for participants. The speed for this test will be determined from the data acquired from sub-maximal running that would reflect a speed corresponding to 85% of maximum heart rate. The calculated speed will be kept constant throughout the test. The initial gradient will be set at 3.5% and increased with increments of 2.5% following the end of each running stage. Furthermore, the duration of each running stage (three minutes) and collection points (final minute of each stage) of expired air, HR and RPE will be kept identical to the sub-maximal testing. The final collection point of data is the point of volitional exhaustion, which is defined as the final minute to be able to sustain a given running speed indicated by the participant. A final minute expired gas sample will be collected at this time point. Satisfactory achievement of $\bar{V}O_{2\max}$ will be fulfilled on the bases of the observation of three of the criteria of the British Association of Sport and Exercise Sciences for establishing maximal oxygen uptake in adult subjects [50]. These include the attainment of a maximal heart rate within 10 beats.min^-1^ from the predicted heart rate maximum for participants (220 beats.min^-1^ - age), respiratory exchange ratio (RER) values of ≥1.1 and subjective indication of volitional exhaustion as indicated by the participant on the RPE scale. The data acquired from these tests can then be used to calculate the treadmill speeds that elicit 60% and 70% $\bar{V}O_{2\max}$, which will be required during warm-up and running procedures in the main trials, respectively.

#### Familiarisation

The participants will be required to complete a familiarisation session at least two weeks prior to their main trials. The familiarisation visit will involve participants completing the main exercise protocol procedures to be performed during the trial (described in the experimental protocol) but without any collection of tissue samples. This visit, therefore, allows participants to familiarise with the study procedures and, thus, diminish any learning/trial-order effects. This visit will also enable us to confirm that the calculated running speeds reflect the required intensity (that is, 60% and 70% $\bar{V}O_{2\max}$) during the trial, with any adjustments to speed deemed by expired gas data being applied accordingly.

### Physiological measurements

#### Urine output

Baseline urine samples will have been obtained 30 minutes before testing to determine hydration via the freezing point depression method by using a cryoscopic osmometer (Advanced Instruments, Inc, Norwood, MA, USA). The threshold for adequate hydration will be assumed for osmolality values ≤900 mOsm.kg^-1^[51]. During the four hour recovery period, the voided urine will be collected in a vessel containing a preservative (5 ml of 10% thymol isopropanol). Upon recording total urine output during this period, a mixed 1 ml sample will be taken and stored at -80°C to enable the estimation of total urinary urea nitrogen excretion, which will subsequently be corrected with plasma urea for whole-body determination of the urea pool during recovery [52]. Non-protein respiratory exchange ratios (NPRER) will also be calculated to reflect protein oxidation rates during the four hour recovery phase [53].

#### Expired gas sampling

Expired gas samples will be obtained via the Douglas bag method (Hans Rudolph, Shawnee, KS, USA) from each participant. A respiratory valve with a mouth piece will be attached to a 200 litre Douglas bag for expired gas sampling, which will be provided with a nose clip to participants approximately 30 seconds before sampling to remove any residual atmospheric air from the valves. The collected gas samples will then be analysed for relative expired fractions of oxygen and carbon dioxide using paramagnetic and infra-red analysers, respectively (Servomex, Crowborough, UK). The total volume of expired gas within the Douglas bag will subsequently be measured by a dry gas meter (Harvard Apparatus, Kent, UK), with the temperature of expired gases being collected at the time of evacuation by a thermistor probe. Prior to any gas collection, calibration of equipment will be conducted using gas cylinders containing specific gases with a known relative composition (N_2_ = 0%; O_2_ = 16.9%; CO_2_ = 4.93%), as validated by the manufacturer (CryoService, Worcester, UK). Each analyser will then be validated against atmospheric air. The inspired air will be measured proximally to the participants throughout the studies, with values used relative to each corresponding expired gas sample to minimise systematic bias associated with assumptions that inspired gas fractions are stable and reflective of atmospheric constants [54].

The calculations of oxygen consumption and carbon dioxide production from each bag will then be used for the determination of carbohydrate and lipid oxidation rates (g.min^-1^) using the following formulae:

$$\begin{array}{l} \text{Carbohydrate}\;\text{Oxidation}=\left(4.212\;\times \;{\text{VCO}}_{2}\right)-(3.005\;\times {\mathrm{VO}}_{2}\;)-(2.449\;\times \;N)\\ \text{Fatty}\;\text{Acid}\;\text{Oxidation}=\left(1.754\;\times {\mathrm{VO}}_{2}\right)-(1.754\;\times \;{\mathrm{VCO}}_{2})-(2.017\;\times \;N) \end{array}$$

Where N is the estimated rate of nitrogen excretion based on urinary/plasma urea [52].

Extramuscular carbohydrate oxidation can then be derived from the difference between whole-body carbohydrate oxidation as determined from indirect calorimetry and intramuscular carbohydrate oxidation methods (overall muscle glycogen degradation rates).

#### Blood sampling

During the familiarisation session, blood samples will have been obtained using the capillary fingertip method by a 3 mm puncture (Accu-Check; Roche Diagnostics GmbH, Mannheim, Germany) applied distally relative to each participant’s fingers. These will be dispensed into microvettes (approximately 25 μl) containing lithium heparin to act as an anticoagulant before being analysed for blood glucose and ketone body concentrations (Optium Xceed; Abbott Laboratories Ltd., Maidenhead, UK). Venous blood samples will have been obtained during the main trials via an indwelling cannula; inserted into an antecubital vein under local anaesthesia (1% lidocaine; Hameln Pharmaceuticals Ltd., Brockworth, UK) and kept patent throughout the trials by flushing with heparin-free isotonic saline (B. Braun, Melsungen, Germany) following each sampling point. Participants will be rested in a semi-supine position for 10 minutes prior to any baseline blood sample. A 10 ml blood sample will be collected during each sampling point before being dispensed into 2 × 5 ml tubes. Each blood sample will first be transferred into a non-anticoagulant collection tube (Sarstedt, Leicester, UK), and left to clot for approximately 45 minutes at room temperature before centrifugation at 2000 × g for 10 minutes at 4°C (Heraeus Primo R; Thermo Fisher Scientific, Loughborough, UK) for serum extraction, which will be stored at -80°C pending analysis for insulin concentration. The remaining 5 ml of each venous blood sample will be dispensed into a different tube containing an anti-coagulant (ethylenediaminetetraacetic acid; EDTA) then immediately analysed for haemoglobin concentrations by using an automated haematology analyser (Sysmex SF-3000; Sysmex Ltd., Wymbush, UK). Thereafter, 3 × 50 μl aliquots of blood will be removed using micro-haematocrit tubes and subsequently centrifuged (Hawksley, Lancing, UK) to obtain haematocrit concentrations. Equations based on heamoglobin and haematocrit values will be utilised to determine plasma volume changes throughout the trials [55]. The remaining EDTA-treated blood will then be spun for centrifugation under 2000 x g for 10 minutes at 4°C for plasma extraction prior to being stored at -80°C for later analysis of plasma glucose, lactate, non-esterified fatty acids and urea.

#### Plasma glucose, lactate, non-esterified fatty acids and urea

For the assessment of plasma metabolites, an automated spectrophotometric analyser (RX Daytona, Randox, Crumlin, Ireland) will be utilised. Prior to each sample analysis a calibration will be conducted and quality control will be checked against the manufacturer’s available standards.

#### Serum insulin

Sera concentrations will be assessed by enzyme-linked immunosorbent assays (ELISA) using a spectrophotometric plate reader. The fundamental principle of all these assays is that the target analyte (the antigen) is recognised with high specificity by antibodies within the human body. The immune system produces antibodies in response to the presence of antigens. These antibodies subsequently recognise and bind to the antigens, and the labeling of the resultant bound antibody forms the basis of this method [56].

#### Muscle biopsy sampling and storage

To minimise discomfort for participants while obtaining muscle samples at essential time points for the aims of the study, three muscle biopsy samples will be obtained during each main trial. In Phase I and during the low sucrose treatment arm, a muscle biopsy sample will be taken at the point of volitional exhaustion following the first exercise bout to identify muscle glycogen depletion levels before the short-term recovery period. Indeed, given that participants had consumed replicate diets 48 hours prior to each main trial and the provision of a standerdised final meal (see standardisation of lifestyle section), and were required to run to volitional exhaustion suggests that muscle glycogen levels are expected to be similar between any of the first exhaustive exercise bouts during the main trials. Based on previously reported data that applied a similar dietary and activity standardisation in addition to comparable exercise protocol procedures, it was shown that muscle glycogen depletion levels were unlikely to vary between trials [38]. Hereafter, a biopsy sample will not be required at this time point during the high sucrose trial. Further support of this is gained through the initial run times to exhaustion obtained from participants tested thus far in Phase I, indicating comparable times to exhaustion (data not shown) and, thus, endorsing the notion that intra-individual pre- and post-exercise muscle glycogen contents were at comparable levels between the trials. The remaining two biopsy samples in the low sucrose treatment will be obtained at the end of recovery and at the point of fatigue during the subsequent run. During the high sucrose treatment, the three muscle tissue samples will be obtained at the end of the recovery period, the time corresponding to fatigue during the low sucrose treatment and at the point of volitional exhaustion during the subsequent run.

It is difficult to ascertain the condition under which muscle glycogen storage and/or endurance capacity may be enhanced in Phase II of the trial. Therefore, three biopsies will be obtained at identical time points between the treatment arms to assess relative glycogen resynthesis rates during recovery and their degradation rates during the subsequent exercise bout. To achieve the aforementioned aims, the biopsy time points will be: following the first exhaustive run, at the end of the four hour recovery period and upon cessation of the subsequent run to exhaustion.

A needle biopsy technique [57] will be used to obtain approximately 30 to 100 mg of wet muscle tissue from the *vastus lateralis*. This specific site was chosen: (1) to present muscle glycogen data that are comparable with the majority of investigations conducted in this area; and (2) because of the large contribution of the quadriceps muscles during treadmill running. Although it was demonstrated that muscle glycogen utilisation is greater in the calf muscles than in the thigh muscles [58], it is clear that substantial depletion of muscle glycogen in the quadriceps is present during treadmill running [38] Thus, measurements obtained from the *vastus lateralis* were assumed to reflect the total contribution of muscle glycogen towards overall metabolism under the assumption that dry muscle mass of both legs was 5% of body mass and that glycogen utilisation of these limbs is represented by the *vastus lateralis* sample. Each muscle sample will be taken from a different 3 to 5 mm skin incision site separated by 2 to 3 cm and applied under a local anaesthetic (2 to 3 ml of 1% lidocaine, Hameln Pharmaceuticals Ltd., Brockworth, UK) while participants were in a semi-supine position. An opposite leg will be used during the second main trial for each participant, where the use of dominant/non-dominant limbs will be counterbalanaced between participants.

#### Muscle biopsy analysis

Once removed from the leg, the muscle tissue will be extracted immediately from the needle biopsy and snap-frozen into liquid nitrogen. The muscle samples will then be removed and placed in a cryogenic vial (Corning, Ewloe, UK) while being maintained in liquid nitrogen where they will be subsequently stored at -80°C pending analysis for muscle glycogen concentrations determined spectrophotometrically [59].

#### Decontamination procedures

Once the wet muscle tissue extraction process has been completed, all equipment used in the muscle biopsy technique will undergo a generic decontamination procedure. All muscle biopsy component parts will be disassembled and cleaned individually with warm water. A wire brush will then be used to remove any organic matter, blood or any other deposits prior to being placed in an Ultrasonic bath containing a solution of 0.3% Sonozyme enzymatic soap for sonification for 10 minutes at 30°C. Once sonified, all equipment will be rinsed with ultrapure water before being immersed in a disinfectant (Milton Sterilising Tablets, Newmarket, UK) for 15 minutes before another rinse cycle with deionised ultrapure water. Finally, each item will be individually placed in a sterilising pouch prior to an autoclave at 121°C for 20 minutes.

#### Subjective measurements

Subjective RPEs will be obtained during all exercise trials by using Borg’s 6 to 20 RPE scale [49], where 6 and 20 range from ‘very very light’ to ‘maximum’ effort. Subjective measurements of stomach discomfort, gut fullness and thirst will be recorded using adapted Borg scales in which scales ranged from ‘no discomfort’ to ‘extreme discomfort’, ‘not full’ to ‘very very full’ and ‘not thirsty’ to ‘very very thirsty’, respectively. To account for any mood disturbances during any experiments, a POMS questionnaire will be completed by each participant prior to commencing any of the trials. This questionnaire has been shown to be a viable tool to detect any mood fluctuations in an exercise setting that may have an influence on performance [60,61].

#### Reliability measurements

To evaluate the error variation and establish sufficient precision of measurement, coefficients of variation (CV = SD/mean × 100) will be assessed for intra-assay and intra-gas variations upon the attainment of data from our procedures.

### Statistical analysis

While the primary outcome measure in these experiments is the rate of muscle glycogen resynthesis/degradation, this variable is highly reliable relative to other outcomes and, accordingly, this consistency results in sample size estimates requiring only four or five participants (thus justifying the use of typically six or eight participants in most studies in this field). It was, therefore, deemed appropriate to base sample size estimates on secondary, less reliable, outcome measures. In this case, exercise capacity exhibits the highest between subject variability and our previous work that contributes directly to the rationale for this study observed a difference between the high sucrose and moderate sucrose plus whey protein supplements under investigation here of 7.5 minutes, with a standard deviation of differences of 5 minutes [21]. Based upon these data, it can be estimated that a sample size of seven would be appropriate even to provide a 90% power to detect such effects on these more variable secondary outcomes using a two tailed paired *t*-test.

A paired *t*-test will be used to compare between treatments for the primary and secondary outcomes of the study (that is, glycogen resynthesis/degradation and run times to exhaustion). A two-way linear mixed model with repeated measures (beverage × time) will be employed to analyse the repeated experimental conditions. Mauchly’s test will be used for sphericity. Where asphericity is assumed, the Greenhouse-Geisser correction will be used for epsilon <0.75 and Huynh-Feldt will be adopted for less severe asphericity. Where significant F values are found a Bonferroni step-wise correction will be employed to determine the location of the variance [62]. Simple summary statistics will be used to inform interesting questions related to certain metabolites (for example, plasma glucose) following each feeding strategy and during the subsequent exhaustive bout. Statistical procedures were performed using commercially available software (IBM SPSS version 20.0, SPSS Inc., Chicago, IL, USA) and significance is set at an alpha level of 0.05. All results will be reported as the mean ± standard deviation (SD) of the mean unless stated otherwise.

## Discussion

It remains to be established whether the provision of different amounts of carbohydrate to restore muscle glycogen during short-term recovery may indeed translate into a dose–response relationship with subsequent endurance capacity. Although a dose–response has been independently observed in muscle glycogen restoration [22] and subsequent endurance capacity [21] following an initial exercise bout, no study has directly examined the associated links between these variables in a single cohort of participants. The aim of Phase I of the trial is to examine these effects.

Furthermore, the mechanism(s) for the potential improvements in the restoration of exercise capacity with protein co-ingestion, in addition to establishing the likely cause(s) of fatigue during a subsequent bout, remain to be elucidated. Noticeably, further studies that mimic human locomotion (that is, running-based studies) investigating the area of post-exercise short-term recovery nutrition need to be conducted. Interestingly, an improvement in subsequent endurance capacity with protein feeding was shown, irrespective of the fact that the glycogen levels were similar to an isocaloric supplement [63]. Albeit in that study, independent groups were used to assess glycogen resynthesis rates and endurance capacity. To our knowledge, no study concurrently explored the aforementioned factors. Therefore, the effects of nutrient intake/composition upon limited recovery and the capacity for repeated exercise requires further investigation. The current project aims to contribute in further understanding of the mechanisms of fatigue and the potential role of muscle glycogen during a subsequent exercise bout following short-term recovery and to establish any mediating effects of different nutrient amounts and/or compositions to enhance recovery of human endurance capacity.

## Trial status

Data collection from Phase I is close to completion. Participant recruitment is ongoing for Phase II coexistent with data collection.

## Abbreviations

CV: coefficient of variance; EDTA: ethylenediaminetetraacetic acid; ELISA: enzyme-linked immunosorbent assays; HR: heart rate; NPRER: non-protein respiratory exchange ratio; POMS: profile of mood state; RER: respiratory exchange ratio; RPE: rating of perceived exertion; TT: time trial; TTE: time to exhaustion; $\bar{V}O_{2}$: oxygen uptake; $\bar{V}O_{2\max}$: maximal oxygen uptake.

## Competing interests

The authors declare they have no competing interests.

## Authors’ contributions

AFA: conception and design, data collection and analysis and manuscript writing. JAB: conception and design, critical revision and data collection and analysis. KT: conception and design, critical revision and data analysis. DT: data collection and critical revision. JB: conception and design and critical revision. All authors read and approved the final manuscript.
